# The impact of legalization of access to recreational Cannabis on Canadian medical users with Cancer

**DOI:** 10.1186/s12913-020-05756-8

**Published:** 2020-10-27

**Authors:** Philippa Hawley, Monica Gobbo, Narsis Afghari

**Affiliations:** 1 Pain and Symptom Management/Palliative Care, British Columbia Cancer, 600 W 10th Ave, Vancouver, BC V5Z 4E6 Canada; 2 Department of Medicine, University of British Columbia, Vancouver, BC Canada

**Keywords:** Cannabis, Cancer, Survey, Symptom management

## Abstract

**Background:**

Canada legalized cannabis use for medical purposes in 1999. Legalization of cannabis for recreational purposes in October 2018 offered the opportunity to assess the impact of recreational legalization on cancer patients’ patterns of use to identify learning points that could be helpful to other countries considering similar legislation.

**Method:**

Two identical anonymous cross-sectional surveys were administered to cancer patients in British Columbia 2 months before and 3 months following legalization, with the same eligibility criteria.

The prevalence of medical cannabis use, the distribution of symptoms leading to use, the most common types of cannabis products and sources, reasons for stopping using cannabis, and barriers to access were assessed.

**Results:**

The overall response rate was 27%. Both cohorts were similar regarding age (median = 66 yrs), gender (53% female), and education (approximately 85% of participants had an education level of high school graduation and higher). Respondents had multiple motives for taking cannabis, including to manage multiple symptoms, to treat cancer, and for recreational reasons. The majority of patients in both surveys did not use the legal medical access system.

Comparison of the two cohorts showed that after legalization the prevalence of current cannabis use increased by 26% (23·1% to 29·1%, *p*-value 0·01), including an increased disclosure of recreational motive for use, from 32 to 40%. However, in the post-legalization cohort more Current Users reported problems getting cannabis (18%) than the pre-legalization cohort (8%), (*p*-value < 0·01). The most common barrier cited was lack of available preferred products, including edibles, as these were only available from illegal dispensaries.

**Conclusions:**

Results showed that legalization of cannabis for recreational purposes may have an impact on those who use medical cannabis. Impacts include an increase in prevalence of use; problems accessing preferred products legally; higher cost, and difficulties using a legal access system. The desired goal of regulation in reducing harms from use of illegal cannabis products are unlikely to be achieved if the legal process is less attractive to patients than use of illegal sources.

## Background

Despite encouraging pre-clinical studies, there is little to no human clinical trial evidence of potential for prolongation of survival in cancers other than some small preliminary studies in glioblastoma [1]. Cannabinoids of a variety of types have however been shown to alleviate several symptoms common among cancer patients [2]. Cancer patients report using cannabis extracts and synthetic cannabinoids to manage their cancer-related symptoms, including pain, sleep disturbance, anxiety, nausea, and anorexia [3–7]. Also, other symptom management drug consumption has been found to be reduced among cancer patients using cannabis products [4]. There is, however little research on the benefits and harms of using cannabis-based products for medical purposes [5].

There are currently two cannabis-based pharmaceutical products available by prescription in Canada to people living with cancer, multiple sclerosis or acquired immunodeficiency syndrome (AIDS), for management of pain, muscle spasm, and anorexia respectively. Nabiximols/Sativex®) is prohibitively expensive, and the synthetic tetrahydrocannabinol (THC) analogue nabilone/Cesamet® lacks any of the non-THC components present in naturally occurring cannabis-based products, particularly cannabidiol (CBD), which has been shown to reduce side-effects of THC, and to have independent effects [6–9].

Canada legalized the use of plant-derived cannabis for medical purposes in 1999 allowing medical cannabis producers to become licensed to sell dried plant product and (later) oil extracts to patients online, with stringent quality standards, including clear labelling of THC and CBD content, and documentation of absence of molds or pesticide residues. The legal products were generally more expensive than via the illegal route and the process of accessing them was quite onerous [10]. Edibles (oral capsules, tablets, or cannabis-containing food products) were not permitted. Though there have been different iterations of the process over the years, patients have always had to obtain a “medical authorization” document from their physician and then register with an online supplier, making a mail-order purchase with a credit card [10]. An alternative access route has always existed via illegal but unprosecuted storefront dispensaries and unlicensed growers. In 2000 the Supreme Court of Canada deemed access to cannabis for medical purposes a human right, which made prohibition of these unregulated suppliers virtually impossible. Failure to enforce laws prohibiting sale of cannabis products by unlicensed providers led to a lucrative and unregulated cannabis supply industry, with no medical oversight, no standards, highly variable quality of product, and inconsistent or inappropriate guidance for medical users.

In response to this legal quagmire, the Canadian government announced in early 2018 that as of October 17, 2018, Canada would be the second country in the world (after Uruguay), to set up a separate legal cannabis access system for recreational use [11–13], which from here on will be referred to simply as ‘legalization’. After the Cannabis Act came into force, sale, possession, production, and distribution of cannabis in Canada have been legal. Recreational dispensaries (storefront and online) were allowed to apply for permission from Health Canada to sell a variety of cannabis-based products to any purchaser 18 years of age or older, but were not permitted to provide medical information to purchasers. The products had to be derived from a Health Canada-approved source, and only dried plant product or oils were to be permitted. Neither medical authorization nor Health Canada registration were required for purchase from recreational dispensaries. To protect public safety, a strict legal framework was implemented to control production, and non-oil extracts and topicals remained illegal until one year after legalization [14–16].

Under the new legislation, only licensed dispensaries were allowed to sell legal recreational cannabis products, and many unlicensed dispensaries were forced to close while their license applications were being processed [15, 17]. At the time of this study, 3 months after legalization, there was only one licensed dispensary open in British Columbia (BC) [18]. Widespread publicity of the planned date of legalization created a unique opportunity to observe the use of cannabis in the medical setting both before and after recreational legalization.

Part I of this study was a survey which was developed and administered roughly 2 months prior to legalization to British Columbia Cancer (BCC) Centre attendees [19]. Results from this survey demonstrated a high usage of cannabis in a variety of forms by cancer patients in BC. Just over half (52%) had used cannabis at some point in their lifetime, and most past users reported their prior goal of use to be recreational. Half of those who had ever used cannabis reported currently taking cannabis, i.e. one in four responding cancer patients reported currently taking cannabis or cannabis-based products. The most common reasons for use were for symptoms: pain (62%), insomnia (41%), nausea (39%), and anxiety (36%); but many (52%) reported also ticked the survey option of taking it for ‘cancer treatment’.

Though 32% of Current Users disclosed some recreational reason for use as well as medical, only 6 of the 190 Current Users (3.2%) stated their use was purely recreational. Current Users tended to primarily use cannabis oil extracts to alleviate multiple symptoms simultaneously, suggesting potential for cannabinoids to substitute for other drugs such as analgesics, anti-emetics, hypnotics, and anxiolytics [10]. These results were consistent with those of similar studies in Canada which reported on cannabis use in the medical setting prior to legalization [20, 21].

The survey was repeated in another cohort of BCC patients randomly selected in exactly the same way, 3 months after legalization, to see if legalization had impacted the prevalence or patterns of use. This report describes the results from the second survey and compares them with the first.

## Methods

**Fig. 1:**
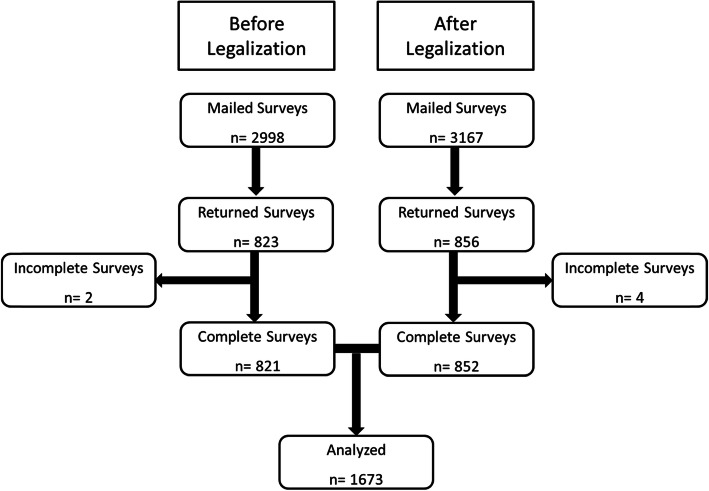
Consort Diagram

The survey and its administration were exactly the same as for the first survey described in detail previously [10]. In brief, the survey was designed to capture cannabis use prevalence and patterns and mailed to all patients who were scheduled for an appointment at any of the six BCC Centres on an arbitrarily chosen study day. As noted in our first report, BCC is a provincial organization and sees 94% of the total number of cancer patients seen for specialist oncology services each year in BC (Fiscal 2017/2018). The surveyed cohort can therefore be considered representative of the cancer patient population in the province, which includes urban, rural, and remote populations. The one-page survey had been reviewed previously for ease of readability by a group of patient volunteers prior to administration (see Fig. 1). The study was approved by the University of British Columbia Research Ethics Board (H18–01638). Eligibility for a cash prize was offered as an incentive for participation, and lottery entries were separated from the surveys immediately upon opening. All surveys were reviewed anonymously. Returned surveys were collected for 1 month following the study day and entered into the REDCap data capturing system [22, 23]. The first and second surveys were differentiated by paper colour to avoid confusion between the two cohorts.

Respondents were asked to state their age; gender; ethnicity; level of education; primary cancer diagnosis (if any); understanding of treatment stage; current and past cannabis use; form(s) used; source of product(s); reason(s) for use; whether they had had any difficulties accessing cannabis; and (if a past user) their reasons for stopping. All information was provided anonymously. The only chart-derived data was the appointment booked on the arbitrarily selected index day (Wednesday January 9th, 2019).

Survey respondents were assigned into user groups (Never, Prior, and Current Users) and within groups into pre- and post-legalization cohorts. Comparisons between groups were made using the chi-square test of independence and two sample *t-*tests.

## Results

**Fig. 2:**
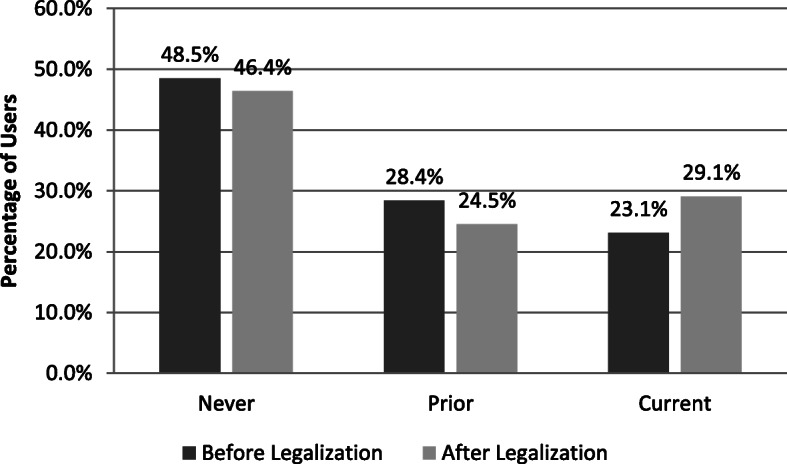
Total Users

The response rate of 27·0% (852 out of 3167 surveys mailed) was almost exactly the same as for the pre-legalization survey mail out; 27·4% (Fig. 2). Current taking of cannabis was reported by 29.1% of participants (*n* = 248) (Table 1). Respondent demographics were very similar between the two survey cohorts. In the second (post-legalization) cohort Never, Prior, and Current Users were all very similar in regard to gender, primary cancer diagnosis, treatment stage, and education. Approximately 85% of participants had an education level of high school graduation and higher. The majority of participants were Caucasians (81%) and Asians (7%). Fifty five percent of Caucasians and 31% of Asians in this study were prior or current cannabis users (Table 2). Current and Prior Users had a lower median age compared to Never Users (62, 64, and 70 yrs., respectively; *p*-value < 0·01).

**Table 1:** Cannabis use before and after recreational legalization in Canada

	Before		After		*p*
	*n* = 821	%	*n* = 852	*%*	
Users (%) ^a^					
Never	398	48·5	395	46·4	0·01
Prior	233	28·4	209	24·5	
Current	190	23·1	248	29·1	

^a^ *p* values were calculated using the chi-square test of independence

**Table 2:** Demographic comparisons of respondents who are Never, Prior, and Current Users of cannabis before and after legalization of recreational cannabis in Canada

	Never Users			Prior Users			Current Users		
	Before	After	*p*	Before	After	*p*	Before	After	*p*
	*n* = 398	*n* = 395		*n* = 233	*n* = 209		*n* = 190	*n* = 248	
Age ^a^									
Median	70	70	0·91	63	64	0·03	61	62	0·54
IQR	61–76	62–77		54–69	56–71		52–70	55–69	
Not Reported	3	3		1	2		1	1	
Gender									
Female	213	217	0·69	127	115	0·86	94	123	0·99
Male	182	175		105	92		95	124	
Other	0	0		0	1		0	0	
Not Reported	3	3		1	1		1	1	
Race									
Caucasian	284	314	0·25	187	181	0·22	155	197	0·90
Asian	49	42		10	8		7	11	
Aboriginal	6	1		4	1		8	8	
East Indian	8	9		0	3		2	2	
African American	1	0		0	1		2	1	
Latin American	2	1		0	1		0	0	
Other	25	12		20	3		11	16	
Not Reported	23	16		12	11		5	13	
Primary Cancer									
Breast	93	102	0·23	58	59	0·72	31	46	0·87
Prostate	59	65		27	32		26	23	
Gastrointestinal	56	42		27	23		26	44	
Blood/Lymph System	52	46		25	23		23	29	
Lung	41	32		21	14		24	24	
Head/Neck	18	20		25	17		14	19	
Gynecologic	28	19		14	9		12	13	
Skin	15	11		14	7		11	14	
Brain	9	9		9	6		7	7	
Genitourinary	7	13		5	4		4	7	
Sarcoma	3	11		3	6		4	8	
Other	13	13		5	6		7	12	
Not Reported	4	2		0	3		1	2	
Treatment Stage									
Newly Diagnosed	12	20	0·47	13	13	0·04	10	8	0·55
In Treatment	254	249		137	140		130	163	
Finished Therapy	100	98		76	44		39	55	
Not Receiving Treatment	30	25		7	12		11	20	
Not Reported	2	3		0	0		0	2	
Education									
Less than High School	20	17	0·23	7	5	0·22	8	9	0·84
Some High School	41	35		17	11		13	21	
High School/GED	72	84		29	35		41	57	
Some College	51	72		59	36		47	51	
College Graduate	105	92		67	73		49	64	
Graduate Degree	104	92		48	45		29	46	
Not Reported	5	3		6	4		3	0	

*IQR* interquartile range

^a^ Age was calculated using a 2-sample *t* test

^b^ *p* values were calculated using the chi-square test of independence and did not include ‘Other’ and ‘Not Reported’ categories

Current Users took cannabis for multiple reasons (Table 3). The most common target symptoms were pain (58%), insomnia (42%), anxiety (36%), and nausea (33%). Recreational use was disclosed by 40% of current users and, nearly half (47%) also used it with a goal of treating their cancer. Prior Users stated their use was most often recreational (58%), with the next most frequent reason being pain (27%).

**Table 3:** Descriptions of cannabis use among respondents who are Prior and Current Users comparing before and after recreational legalization of cannabis in Canada

	Prior Users			Current Users		
	Before	After	*p*	Before	After	*p*
	*n* = 233	*n* = 209		*n* = 190	*n* = 248	
Medical Authorization						
No	199	183	0·48	128	178	0·35
Yes	32	24		58	66	
Not Reported	2	2		4	4	
Product ^a^						
Smoked	174	141	0·81	105	159	0·92
Oils	75	75		139	174	
Eating	83	63		86	119	
Vaporized	14	11		61	82	
Cream	21	13		46	71	
Tablets	10	10		24	29	
Drinking	3	2		16	25	
Mouth Spray	3	1		9	12	
Suppositories	4	1		6	14	
Other	6	6		16	16	
Not Reported	0	5		0	0	
What do you use it for? ^a^						
Recreational Use	152	121	0·78	60	99	0·79
Pain	51	57		118	143	
Cancer Treatment	25	30		99	116	
Insomnia	31	28		77	104	
Nausea	30	25		74	81	
Anxiety	22	20		69	89	
Lack of Appetite	16	12		59	68	
Depression	11	7		32	48	
Tiredness	10	6		24	36	
Drowsiness	3	1		16	18	
Other	7	5		15	12	
Not Reported	0	4		0	0	
Where do you get it from? ^a^						
From a friend	171	143	0·33	85	134	0·40
Dispensary	46	52		114	132	
Licensed Producer	13	16		28	41	
Grow it myself	4	7		17	26	
Other	15	12		11	17	
Not Reported	5	5		0	3	
Problems in getting Cannabis?						
No	184	164	0·64	172	201	< 0·01
Yes	11	12		15	44	
Not Reported	38	33		0	3	

^a^ *p* values were calculated using the chi-square test of independence and did not include ‘Other’ and ‘Not Reported’ categories

One of the most important findings of this survey was that the majority of current users did not use the legal online system set up for patients to access medical cannabis, despite using it for medical (*n* = 149) rather than recreational use (*n* = 49). Only 27% (*n* = 66) of Current Users had a medical authorization. This was further broken down to be 30% of current users who reported using cannabis only for medical purposes and 21% of current users who reported using cannabis at least partly for recreational purposes.

**Fig. 3:**
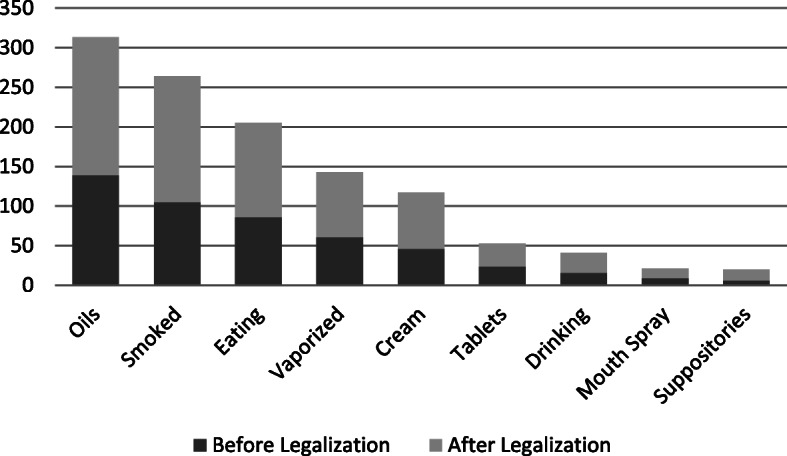
Modes of Administration (Products) of Cannabis

Current Users obtained their cannabis from a friend (54%) and from illegal dispensaries (53%) almost equally. Current Users reported using multiple forms of cannabis products, but used oils the most (70%) followed by smoking (64%), eating (48%), vaporizing (33%) and topically (29%) (Fig. 3). Current users were asked if they had problem getting access to cannabis products. Qualitative data were collected through open-ended questions. Forty-four out of 248 Current Users (17.7%) reported that they had problems getting cannabis, and 41 provided qualitative comments. Three main themes were identified. Sixteen respondents reported specific products were not available, 15 reported having access issues access with dispensaries, and 5 disclosed problems in getting medical authorization. Participants’ representative quotes are summarized in Table 4.

**Table 4:** Current Users’ representative quotes on problems in getting access to cannabis after legalization

Theme	Representative Quotes	Frequency	Proportion
**Specific items from dispensaries not available**	- -Very hard to get CBD oil.	- 16	- 0.36
	- -The removal of CBD and topicals from the dispensary is stupid and cruel and harming people who need it.		
	- -Since cannabis has been legalized it is impossible to get certain products, or preferred brands.		
	- -Suppositories not legal and won’t be in 100 mg doses when it becomes legal.		
	- -Other than the gummies I can’t get anymore, so I switched to oil.		
	- -Hard to source good edibles with consistent dosage.		
	- -Hard to find eatables.		
	- -Hard time getting stronger dosages.		
	- -Getting CBD was hard at first, everyone was producing THC now it is easy to get.		
	- -Edibles were banned from dispensaries, plus tonics.		
	- -Edibles used to be available but now have stopped.		
	- -Edibles (chocolates, etc.) no longer available; I make my own using oils.		
	- -Do not smoke, quit over 2 years ago. Would use edibles more if they were legal and more easily available. Use oil if available. Know a person that has medical, but doctors not get anymore.		
	- -Because I can’t smoke it anymore and edibles are illegal.		
	- -A little, edibles should be legalized now.		
	- -January 2019 - no legal access in my city for legal drug! I can’t find edibles and that’s what I want		
**Issues with dispensaries and access to products**	- -Increased demand = decreased supply inconvenience of many dispensaries not licensed, Lack of access to licensed provider /lack of access to standardized product need/waiting high dose CBD difficult to find.	- 15	- 0.34
	- -Slow delivery.		
	- -The dispensary in my residential neighborhood is closed - inconvenient to go to another location for it.		
	- -With licensing, reliable distributors closed down and sub-par, criminal-run dispensaries were left open only since recreational use was legalized!!! Prior to legalization I had 3 good sources in my city but they were forced to close. I traveled to Kamloops to get some product but they were out of what I needed. There should be some special need recognized for the use of medical cannabis! There was no problem with quality of the cannabis I use for medical purposes - nobody refines that stuff in their basement. I tried ordering it online and that was an exercise in futility! We NEED better access to it for medical use. And be more affordable. Thank you. (I will not give my $ to these establishments).		
	- -Legally, not enough access.		
	- -Licensed producer (BC Gov) often has supply issues.		
	- Initially I was able to buy the THC Pheonix tears through a dispensary. After it was legalized the dispensary closed + I then had to source it from a friend who makes it for himself. He had lymphatic cancer and used only -THC he is now cancer free.		
	- -No dispensaries in Prince George.		
	- -Due to the onerous, cumbersome, and poorly thought out method of licensing, all the local dispensaries have closed.		
	- -Legalization has prevented all dispensaries I used from re-opening - apparently due to local city council morons. Perfectly good system of dispensaries destroyed by political morons abusing their powers at local level since legalization.		
	- -Licensing regulations in my area have caused closure of stores and none have re-opened yet.		
	- -Legal cannabis stores still not open.		
	- -Easy to get on the street - cannot get a retail.		
	- -Dispensary unable to sell to me now! Why is that??? Where can one purchase?		
	- -I live in northern BC = medical dispensary used to mail out, now since legalization they don’t = access down, cost increases		
**Issues in getting medical authorization**	- -Trouble getting authorization.	- 5	- 0.11
	- -Need Dr’s note.		
	- -Medical form requires re-authorization every 6 months and has been struggle to get oncologist to sign and submit. It is January now and still waiting for request since October.		
	- -Since cannabis has been legalized it is so difficult to get a general practitioner, or arthritis specialist to give you a prescription, and otherwise you need to pay some cannabis doctor for a prescription and it is cost prohibitive cost wise for us low income patients.		
	- -Legal restrictions.		
**Issues with cost**	- -It is very expensive.	- 3	- 0.07
	- -Friends not always reliable, outlets are too expensive.		
	- -200 mg suppositories are $7 each ×3 daily is too much $		

For patients reporting prior but not current use, the prior use was often many years previously and had been primarily recreational in intent (58%). Respondents had stopped because of losing interest; safety concerns; or not having used it since their youth (no specific reason stated). Some had tried it for management of cancer-related symptoms, particularly pain (27%) and had discontinued it, most often due to lack of effectiveness, but some stopped because of side effects (Table 5).

**Table 5:** Reasons respondents who are Prior Users ceased cannabis use

	Prior Users		
	Before	After	*p*
	*n* = 233	*n* = 209	
Why did you stop? ^a^			
Not effective	31	42	0·69
Intolerable Side-Effects	26	25	
Cost	27	19	
Advice from Doctor	9	7	
Safety Concerns	33	25	
Other:	3	4	
Difficult to access	13	22	
No longer needed	4	5	
Contraindication	18	18	
Lost interest	29	35	
Not since youth (20+ years ago)	18	20	
Did not enjoy it	46	15	
Not Reported	18	9	

^a^ *p* values were calculated using the chi-square test of independence and did not include unspecified ‘Other’ and ‘Not Reported’ categories

### Impact of legalization

There was an increase in respondent disclosure of Current Use of cannabis from 23·1% in the pre-legalization cohort to 29·1% in the post-legalization cohort (*p*-value < 0·01). No significant differences were found among the demographics of groups when comparing pre- and post- legalization. Before legalization, 32% of Current Users disclosed some recreational motivation for use. After legalization this increased to 40%, including 12 out of 248 (4.8%) who stated their motivation for taking it was purely recreational. Thus, among Current Users, 68% before legalization and 60% after legalization had purely medicinal motives for taking cannabis products. This difference was not statistically significant (*p*-value > 0.05). There were no changes in medical authorization prevalence, products used, symptom-related reasons for use, or where cannabis was obtained from before to after recreational legalization of cannabis.

After legalization, Current Users reported more problems getting cannabis (18% post-legalization compared to 8% pre-legalization, p-value < 0·01) with the most common barriers cited as lack of available dispensaries (*n* = 19), lack of preferred product (*n* = 15), and cost (*n* = 4) (Table 6).

**Table 6:** Reasons Prior and Current Users had problems obtaining cannabis

	Prior Users			Current Users		
	Before	After	*p*	Before	After	*p*
	*n* = 11	*n* = 12		*n* = 15	*n* = 44	
Cost	1	1	0·33	2	4	0·30
Closed Dispensaries/No available legal access	4	3		2	19	
Difficulty getting medical authorization	2	1		2	3	
Specific products (e.g. edibles) or strengths unavailable	0	3		3	15	
Other	2	1		2	0	
Not reported	2	3		4	3	

^a^ *p* values were calculated using the chi-square test of independence and did not include ‘Other’ and ‘Not Reported’ categories

## Discussion

### After legalization

Our results showed that the most common target symptoms patients reported taking cannabis products to help with were pain (58%), insomnia (42%), anxiety (36%), and nausea (33%). The results of this study are strikingly similar to two 2019 reports from the US. One report described a retrospective review of ambulatory palliative care clinic patients from New Hampshire and Vermont, and found a 27% current cannabis use rate, primarily for medical purposes. Patients were often treating multiple symptoms: pain (59%), anorexia (19%), insomnia (17%), nausea (16%), anxiety (10%) and depression (6%) [24]. It would be expected that a palliative care population would be more likely to be symptomatic than our unselected cancer centre-attending population, and therefore be more likely to use medical cannabis, yet our study reported the same prevalence and reasons for use. Very similar results were reported in an ambulatory Seattle cancer patient study [25].

Our results showed that Current Users often took multiple forms of cannabis products, but used oils the most (70%) followed by smoking (64%), eating (48%), vaporizing (33%) and topically (29%). Consistent with our findings, a similar US study of cancer centre patients showed that the prevalence of using cannabis via inhalational and oral routes were equal [25].

Prior Medical Users reported multiple reasons for stopping taking cannabis, including ineffectiveness; intolerable side effects; and safety, and were slightly older (64 yrs) than Current Users (62 yrs). A 2017 systematic review reported that cannabinoids were less effective in managing chemotherapy side effects in older patients than in younger patients [5]. Participants in clinical trials using fixed doses of cannabis-based products also consistently report multiple side effects [5]. Our results with respect to side-effects and stopping taking cannabis are consistent with these studies.

The similarity in results between studies conducted in the US and Canada suggest that other countries considering legalization can expect similar patterns.

### Comparison between before and after legalization surveys

Comparing our two surveys, we found that legalization was associated with a 26% increase in the prevalence of current cannabis use, from 23·1% to 29·1% (*p* = 0·01). One explanation for this increase might be that in the run-up to legalization news and media outlets were filled with articles about dispensaries opening and closing, [18, 26, 27] products available, [28, 29], and new research [30–33], which may have emboldened more patients to try cannabis. Despite both surveys being anonymous, they may also have felt more comfortable disclosing recreational motivation for use.

Though there are places where both medical and recreational access to cannabis are legal, we are not aware of any data from other jurisdictions legalizing access to recreational cannabis in the setting of a separate established medical supply system.

Despite the increase in current users, the choice of cannabis products and reasons for taking them remained much the same between both surveys. The high frequency of reporting use for multiple symptoms strengthens the suggestion that cannabis products should be further studied for potential to reduce polypharmacy in symptomatic cancer patients. Use of cannabis as a form of cancer treatment also remained one of the most common reasons for cannabis use between surveys, and is concerning considering the lack of good clinical trial evidence for any survival benefit from cannabinoid use in cancer.

Our survey reported that only one third of current users who reported using cannabis for medical purposes had the medical authorization necessary for accessing the official medical access system.

Our study identified some problems that impacted medical users immediately following recreational legalization. We found that unlicensed dispensaries and other illegal sources were much more commonly used than the legal medical system, despite their lack of reliable labelling and absence of quality control. The results showed an increase in the prevalence of current cannabis use and also in problems accessing medical cannabis after legalization.

There were multiple reported barriers to use of the legal medical access system, reported by both survey cohorts. Patients who wished to buy high quality legal medical cannabis had to negotiate the online system of access, including getting a medical authorization and waiting for processing of their registration. Respondents from the second survey identified the lack of legalization of certain products (primarily edibles), as their reason for preferring to continue to use illegal sources. Other reported barriers included the need to have a credit card and a stable address for delivery of product, and also difficulties in deciding what to order without the benefit of face-to-face interaction with a salesperson. Medical users’ access to appropriate products may also have been impeded by the fact that staff in recreational dispensaries are prohibited by law from providing medical advice.

The complexities of having two different licensing systems, and confusion between medical and recreational use made it difficult for patients and health care providers to figure out where to access reliable information and product suitable for medical purposes. It should be mentioned that BC was not unique in this respect in Canada [17, 34].

Similarly, respondents from the second survey identified the lack of legalization of certain products (primarily edibles), as their reason for accessibility barrier.

### Implications and suggestions

Other jurisdictions planning to legalize recreational cannabis should consider the impact it might have on medical cannabis users. Medical and recreational use of cannabis often overlaps, at least in cancer patients, and it is concerning that many patients report believing/hoping that cannabis might help treat their cancer. Though much clinical research remains to be done, there is sufficient information available from credible sources (e.g. Health Canada) about doses, indications for medical use, and potential harms for appropriate guidance be created that should be made available in all vendor locations, irrespective of vendor focus.

### Strengths and limitations

Results of this study provide new insights into cannabis use among cancer patients in British Columbia. This study is the first to provide data on the impact of recreational legalization on medical users. Strengths of this study include the close comparability of characteristics among participants in both cohorts. Open-ended questions in the survey allowed participants to share their experience with regards to barriers in obtaining cannabis, which added depth to understanding of the data.

As with all surveys, those that responded to the survey may not be representative of the surveyed population, thus our 27% response rate could reflect a sampling bias. The close matching of the demographics of respondents to the two consecutive surveys however gives us confidence that the differences demonstrated between the two cohorts’ responses reflect actual change. As the data was collected by self-report, and despite the surveys being anonymous, there may have been more openness in reporting recreational motives for cannabis use in the second cohort, with legalization of recreational use reducing stigma. There may also have been a recall bias about respondents’ prior cannabis use.

Another limitation is that there was no control group, and it is possible that the differences seen between the two cohorts may have been due to factors other than recreational legalization. The time difference was however only 5 months.

Though unlikely to have been a major confounder, there was potential for confusion about the definitions of routes of ingestion between our two survey cohorts that we were not aware of at the time the surveys were designed and tested by our patient partners. At the time of the post-legalization survey, gel capsules filled with oil were just becoming available legally and could possibly have been classified by some respondents as “edibles” rather than oils, whereas most “edibles” available illegally to the pre-legalization cohort were in the form of cookies, brownies and candies which were (and remain) illegal. Also, legally obtained oils could conceivably have been compounded for topical use by the respondents.

## Conclusions

This study adds to the literature on medical cannabis use in cancer patient populations and suggests that legalization of recreational cannabis in Canada had an effect on medical users, where there was a separate established safe medical supply system. Our results suggest a correlation between increased cannabis use in cancer patients and legalization of recreational cannabis, as many patients using cannabis-based products for medical purposes access them through routes outside the official medical access process. Separating medical and recreational access programs is not important for many patients, and making sure that legal access routes have clear advantages for patients over existing illegal sources will be crucial to success for any legalization initiative. Product range, price, face to face contact with vendors, and complexity of access all need to be considered if illegal supply systems are to be eliminated and the benefits of regulation are to be realized.

## Data Availability

The datasets used and/or analysed during the current study are stored on a secure database at BC Cancer and are available from the corresponding author on reasonable request.

## Abbreviations

AIDS: Acquired immunodeficiency syndrome
BC: British Columbia
BCC: British Columbia Cancer
CBD: Cannabidiol
IQR: Interquartile Range
THC: Tetrahydrocannabinol
US: United States
